# Nutrient advantage of karst peaks cluster depressions and plant diversity in response

**DOI:** 10.3389/fpls.2026.1786223

**Published:** 2026-02-25

**Authors:** Jianli Zhang, Xuemin Tang, Lihua Pu, Yunjie Wu, Weiquan Zhao, Yang Cao

**Affiliations:** 1 College of Eco-Environmental Engineering, Guizhou Minzu University, Guiyang, China; 2 Guizhou Institute of Mountain Resource, Guizhou Academy of Sciences, Guiyang, China

**Keywords:** karst peak cluster depression, nutrient advantage, nutrient elements, plant diversity, stoichiometric ratio

## Abstract

**Introduction:**

The karst peak-cluster depression ecosystem is ecologically fragile, and the relationship between vegetation diversity and soil nutrients is a key scientific issue for ecological restoration. However, the relationships among soil chemical composition, nutrient elements, physicochemical properties, and plant diversity indices across different vegetation types, as well as the pathways through which key factors influence each other, remain unclear.

**Methods:**

This study investigated grasslands, shrublands, and forests in Pingtang County, Qiannan Prefecture, Guizhou Province. Using correlation analysis and partial least squares structural equation modeling (PLS-SEM), we examined the associations and driving factors linking soil physicochemical properties, nutrient elements, mass ratios, and plant diversity.

**Results and discussion:**

We found that: (1) Shrubland vegetation exhibits significantly higher soil organic carbon (SOC) content than grasslands and forests, indicating greater carbon sequestration potential; total nitrogen (TN) was the primary limiting nutrient. (2) Shrubland communities reached peak values for Margalef, Shannon-Wiener, and Pielou indices, with SOC showing a significant positive correlation with the Pielou index. (3) Soil organic carbon (SOC), total nitrogen (TN), and total phosphorus (TP) content were core factors regulating plant species diversity. The soil nitrogen-to-phosphorus (N:P) ratio was a key regulator, significantly influencing species composition and spatial distribution. (4) The PLS-SEM model further quantified the interaction pathways among these factors, providing a theoretical basis for optimizing vegetation diversity and managing soil nutrients in regional rock desertification control.

## Graphical Abstract

**Figure:**
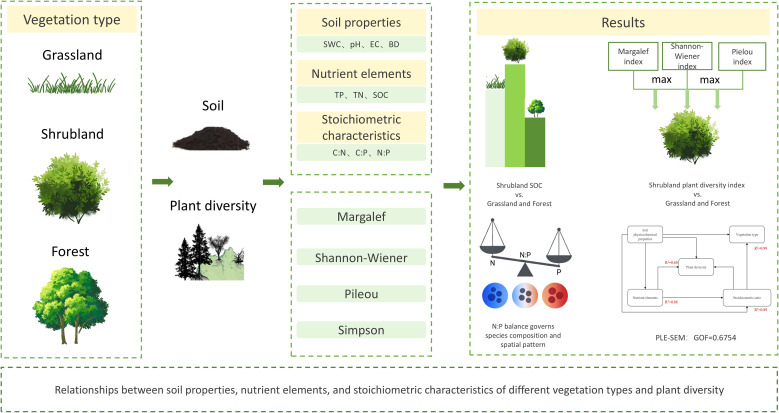

## 1 Introduction

Soil nutrients are pivotal to ecosystem structure and function, regulating aboveground productivity and community composition. For plants, carbon (C) serves as the primary raw material for photosynthesis, nitrogen (N) constitutes proteins and nucleic acids within plant tissues, and phosphorus (P) plays a crucial role in plant growth and reproduction. Soil stoichiometry (C:N:P) is a key indicator for assessing soil productivity and nutrient dynamics (Kumar et al., 2021). Soil stoichiometry primarily investigates the proportional relationships among soil organic matter, soil carbon (C), nitrogen (N), and phosphorus (P), revealing the functions of soil ecosystems. The C:N ratio is a key indicator for measuring soil nitrogen mineralization capacity (Ren et al., 2024), while the N:P ratio is an important indicator of growth rate and nutrient limitation (Pang et al., 2021).

Plant diversity forms the foundation for community formation and stability. Different tree species exhibit distinct biological characteristics that significantly influence soil nutrients. Among soil physicochemical properties, pH, bulk density, moisture content, and electrical conductivity directly affect nutrient forms, availability, and retention capacity, creating the environmental basis for nutrient advantage. The abundance dynamics of plant communities fluctuate with soil nutrient status and its proportional variations (Zhang et al., 2019). Soil quality is recognized as a primary factor controlling plant diversity indices, directly influencing community composition and structure (Zhang et al., 2023). Low bulk density and high porosity promote nutrient release, benefiting plant growth. Recent studies have advanced our understanding of the relationship between plant diversity and biogeochemistry. Studies on species diversity under *Pinus massoniana* plantations and soil physicochemical properties revealed substantial correlations between N and P elements and understory plant diversity indices, although distinct patterns emerged across different ecosystems (Wang et al., 2018). Previous studies have primarily focused on the dynamics of plant and soil ecometrics during succession. Li et al. found that species succession can alter plant community structure by reducing growth-limiting resources and soil microbial diversity (Ning et al., 2021). Research indicates that elevated C:P and N:P ratios in soil can increase the biomass of understory vegetation in forest ecosystem (Fan et al., 2015). Thus, C, N, and P serve as pivotal links connecting species diversity, plant functional traits, and ecological stoichiometry, playing crucial roles in ecosystem processes. Their cycling and balance are particularly sensitive and vital in ecologically fragile regions.

The Southwest China Karst is an extremely fragile ecosystem, characterized by large altitudinal gradients and complex landscape patterns. Alongside loess, desert, and cold desert ecosystems, it is one of China’s four most fragile environmental systems. Rocky desertification, a typical form of land degradation in karst regions, results from severe soil erosion, extensive bedrock exposure, decreased soil productivity, and desert-like landscapes (Sheng et al., 2015; Sheng et al., 2018). Peak clusters and depressions, typical landscapes of rocky desertification, are concentrated in the southern part of China's southwestern karst region. This region exhibits profound rock desertification, limited ecosystem regulatory functions, and pronounced spatial heterogeneity in habitat conditions, posing significant challenges for natural vegetation restoration. As rock desertification progresses, plant diversity in karst habitats declines, community structures simplify, and vegetation often evolves into types dominated by a single dominant species. While numerous studies have examined the relationship between soil ecological stoichiometry and plant diversity indices, most focus on the landscape scale, with limited exploration at the community level across different vegetation types. The relationships among soil ecological stoichiometry, nutrient elements, physicochemical properties, and plant diversity indices across different vegetation types in karst peak clusters and depressions in southwest China, as well as the path characteristics of key influencing factors among them, remain unclear. Therefore, this study aims to (1): investigate the relationships among soil physicochemical properties and nutrient elements across different vegetation types (2); determine the relationships between soil ecological stoichiometry and plant diversity indices across different vegetation types; and (3) quantify the complex relationships among soil physicochemical properties, soil nutrient elements, ecological stoichiometry ratios, and plant diversity indices across different vegetation types; (4) identify primary nutrient limiting factors for grassland, shrubland, and forest vegetation.

We employed a spatio-temporal substitution method to study the area surrounding the Five-hundred-meter Aperture Spherical Telescope (FAST). Three typical vegetation types: grassland, shrubland, and forest, were selected. Soil samples were collected at three different depths: 0–10 cm, 10–20 cm, and 20–30 cm. The basic physicochemical properties of the soil were measured: pH, bulk density (BD), electrical conductivity (EC), and soil water content (SWC). Soil nutrient elements measured included total organic carbon (SOC), total nitrogen (TN), and total phosphorus (TP). The relationships between soil factors and plant diversity indices were analyzed to provide a scientific basis for ecological conservation and restoration in karst peak cluster depression ecosystems.

## 2 Materials and methods

### 2.1 Study area

**Figure 1:**
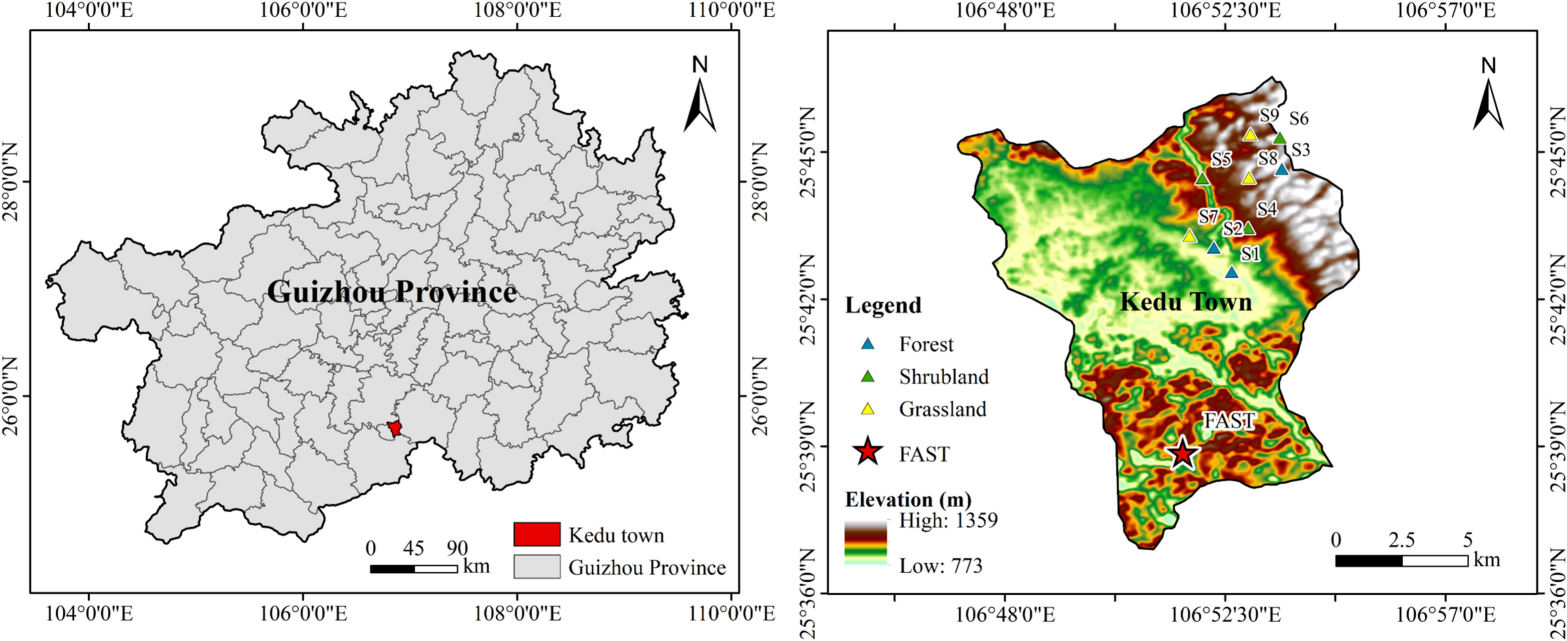
The location of the study area.

The study area is located in Kedou Town, Pingtang County, Qiannan Prefecture, Guizhou Province. Situated at the transition zone between the southeastern slope of the Yungui Plateau and the Guangxi Hills. The geographical coordinates are 106°52’02’’-106°53’37’’ E, 25°43’03’’-25°45’13’’ N. It represents a typical karst peak cluster depression region (**Figure 1**). The area suffers from severe rocky desertification, with shallow and unevenly distributed soil layers. It covers 2,815.60 km², with elevations ranging from 863 to 1,174 meters. The annual average temperature is 15-17 °C, and annual precipitation averages 1,200-1,300 mm. Karst topography accounts for 87.2% of the land area. Limestone is widely distributed throughout the study area, where soil pH is predominantly neutral. The region experiences a subtropical monsoon climate influenced by the Indian Ocean monsoon. The dominant tree species in the study area are *Pinus massoniana*, *Quercus acutissima*, and *Quercus fabri*. Major shrub species include *Gossypium hirsutum L*, *Pyracantha fortuneana, Magnolia denudata Desr*, and *Sichuan pepper*. Key herbaceous plants include *Ficus carica*, *Portulaca oleracea L*, and *Diplazium lanceum*.

### 2.2 Vegetation data collection and analysis

Vegetation surveys employed the plot method, with 20 m × 20 m plots established for grassland, shrubland, and forest vegetation types. Each plot is configured with 10 subplots measuring 4 m × 10 m for tree species, 10 subplots measuring 5 m × 5 m for shrub species, and 5 subplots measuring 5 m × 5 m for herbaceous species. Within each plot, data on plant species, density, cover, and frequency were recorded. The importance value (*IV_i_*) of plant species was calculated to measure dominant species.

The IVi formula is as follows (Awoke and Mewded, 2019; Han et al., 2023):


$$IV_{i}=\left(\frac{D_{i}}{\sum^{}D}\right)+\left(\frac{F_{i}}{\sum^{}F}\right)+\left(\frac{C_{i}}{\sum^{}C}\right)\times 100$$

*IV_i_*: importance value of the *i-th* species, *D_i_*: relative density of the *i-th* species, *F_i_*: relative frequency of the *i-th* species, *C_i_*: dominance index of the *i-th* species.

The Shannon-Wiener index (*H*), Margalef index (*R*), Pielou index (*J*), and Simpson index (*S’*) were employed to reveal the structural characteristics of the community.

The calculation formulas are as follows (Chen et al., 2025):


$$H=-\sum_{i=1}^{S}P_{i}\times lnP_{i}$$

R=(S−1)/lnN

J=H/lnS

$$S'=1-\sum_{i=1}^{S}P_{i}^{2}$$

$$P_{i}=N_{i}/N$$

*S*: number of species within each plot, *N*: total number of plants within each plot, *N_i_*: number of individuals of the *i-th* species, *P_i_*: proportion of individuals of the *i-th* species relative to the total number of individuals in the community.

### 2.3 Soil sample collection and analysis

Based on field vegetation surveys, soil samples from three typical vegetation types: grassland, shrubland, and forest were selected as research subjects. Three plots were established within each vegetation type, with three evenly spaced sampling points in each plot. A total of nine soil sampling points were established, namely Grassland: S_1_, S_2_, S_3_; Shrubland: S_4_, S_5_, S_6_; Forest: S_7_, S_8_, S_9_. At each sampling point, undisturbed soil was collected using a sampler at three depths: 0–10 cm, 10–20 cm, and 20–30 cm. Three replicates were taken at each point, yielding a total of 81 undisturbed soil samples. Collected soil samples were air-dried in a cool, dry location, then cleaned to remove plant roots and gravel. The dried soil was ground and passed through a 0.25 mm sieve for subsequent analysis.

SOC was measured using the potassium dichromate oxidation external heating method, TN by the Kjeldahl method, and TP by perchloric acid–sulfuric acid digestion followed by molybdenum-antimony colorimetry (Wei et al., 2023). Soil pH was measured using the potentiometric pH titration method (3:1 soil to water ratio), while EC was determined with a conductivity detector (5:1 soil to water ratio). Each soil sample was analyzed in triplicate to calculate soil C:N, C:P, and N:P ratios.

Soil moisture content and bulk density were measured using the ring knife method, with calculation formulas as follow (Wang et al., 2021):


$$SWC=\frac{M_{2}-M_{1}}{M_{1}}\times 100\%$$

$$BD=\frac{M_{4}-M_{3}}{V}$$

*SWC*: soil water content (%), *M_2_*: soil fresh weight, *M_1_*: soil dry weight, in grams, *BD*: soil bulk density (g·cm^-3^), *M_3_*: mass of circular ring tool (g^-1^), *M_4_*: total mass of ring knife and dry soil weight after drying (g^-1^), V: volume of ring knife (100 cm^-3^).

### 2.4 Data analysis

Partial least squares structural equation models (PLS-SEM) were constructed using the plspm package in R 4.4.3. One-way ANOVA and LSD (Least Significant Difference) tests were employed to compare differences in plant community species diversity indices, soil physicochemical properties, nutrient elements, and stoichiometry among different vegetation types. Pearson correlation analysis examined interactions among indicators. Data are presented as mean ± standard error. Duncan’s multiple range test was used for significance analysis. Data were processed in Microsoft Excel 2016 and IBM SPSS, and graphs were created using Origin 2024.

## 3 Results

### 3.1 Soil physicochemical properties and nutrient element analysis

**Figure 2:**
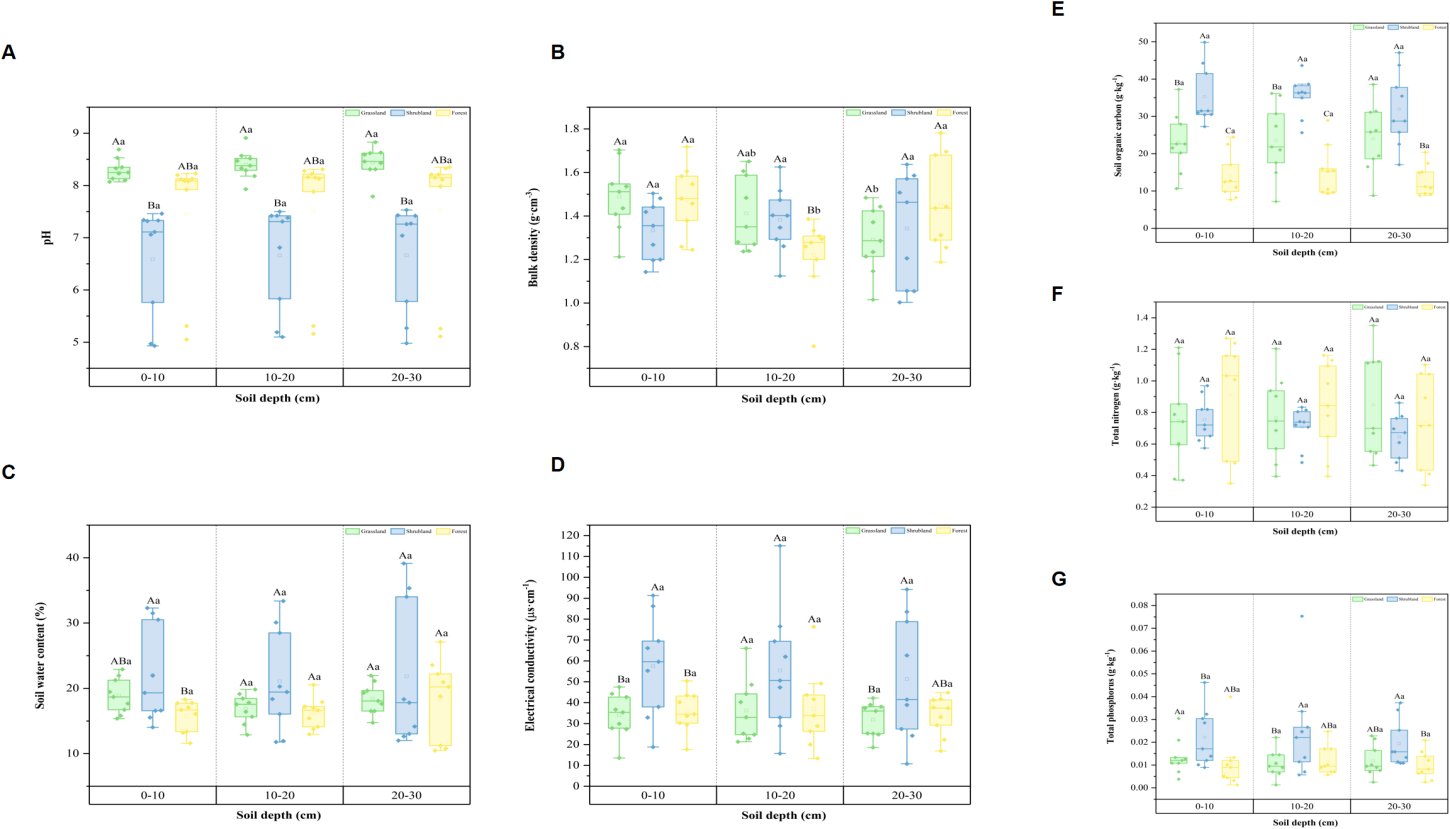
Physical and chemical property characteristics of soil under different vegetation types. Data are mean standard error. Different capital letters indicate significant differences in different vegetation types of the same soil layer (*P<0.05*). Different lowercase letters indicate significant differences among different soil layers within the same vegetation type (*P<0.05*). **(A–G)** represent the effects of pH, bulk density, soil water content, electrical conductivity, soil organic carbon, total nitrogen, and total phosphorus in different vegetation types and soil layers.

Soil physicochemical properties and nutrient analysis for different vegetation types (**Figure 2**). The mean pH values for the 0–30 cm soil layer across the three vegetation types ranged from 6.59 to 8.44; BD ranged from 1.22 to 1.49 g·cm^-3^; mean SWC ranged from 15.75 to 22.03 %; EC ranged from 31.84 to 57.52 μs·cm^-1^; SOC ranged from 13.00 to 35.45 g·kg^-1^; TN ranged from 0.64 to 0.91 g·kg^-1^; and TP ranged from 0.01 to 0.02 g·kg^-1^.

There were no significant differences in pH, BD, SWC, EC, SOC, TN, and TP at different soil depths within the grassland community *(P>0.05*). Differences in pH, BD, SWC, EC, SOC, TN, and TP content at different soil depths in the shrubland community were not significant (*P>0.05*). Differences in pH, SWC, EC, SOC, TN, and TP content at different soil depths in the forest community were not significant (*P>0.05*). Notably, BD content differed significantly between the 0–10 cm and 20–30 cm soil layers compared to the 10–20 cm layer (*P<0.05*).

The maximum EC values in grassland, shrubland, and forest communities all occur in the 10–20 cm soil layer.

### 3.2 Effects of different vegetation types on soil stoichiometric characteristics

Stoichiometric ratios are presented in **Table 1**. Shrubland soils exhibited significantly higher C:N ratios than forest soils (*P<0.05*). Among these, shrubland soils had the highest C:N ratio (51.83), while forest soils had the lowest C:N ratio (23.14). No significant differences in C:P ratios were observed across vegetation types or soil depths. Both grassland and shrubland soils exhibited significantly lower N:P ratios than forest soils (*P<0.05*). Grassland soils had the lowest N:P ratio (1.54), followed by shrubland soils (1.28), while forest soils maintained the lowest N: P ratio (5.21).

**Table 1:** Stoichiometric ratio characteristics of different soil layers with different vegetation types.

Stoichiometric ratio	Vegetational type	Soil layer (cm)		
		0-10	10-20	20-30
C:N	Grassland	35.54 ± 5.97ABa	34.25 ± 5.46ABa	29.88 ± 3.57Ba
	Shrubland	47.62 ± 3.38Aa	51.34 ± 5.84Aa	51.83 ± 7.52Aa
	Forest	23.14 ± 7.43Ba	22.43 ± 5.37Ba	22.97 ± 5.64Ba
C:P	Grassland	41.77+4.72Aa	43.48 ± 6.96Aa	43.85 ± 7.55Aa
	Shrubland	60.16 ± 15.64Aa	60.16 ± 17.41Aa	60.86 ± 17.40Aa
	Forest	68.13 ± 9.08Aa	81.10 ± 15.48Aa	73.84 ± 10.32Aa
N:P	Grassland	1.56 ± 0.34Ba	1.54 ± 0.31Ba	1.60 ± 0.26Ba
	Shrubland	1.28 ± 0.35Ba	1.39 ± 0.41Ba	1.52 ± 0.56Ba
	Forest	6.05 ± 1.49Aa	5.62 ± 1.41Aa	5.21 ± 1.17Aa

Data are mean ± standard error. The ratios C:P, C:N, and N:P are dimensionless. Different capital letters indicate significant differences in different vegetation types of the same soil layer (*P<0.05*). Different lowercase letters indicate significant differences among different soil layers within the same vegetation type (*P<0.05*).

### 3.3 Analysis of species diversity indices in plant communities of different vegetation types

Diversity indices are shown in **Table 2**. No significant differences in diversity indices were observed between vegetation types, except for the Pielou index, which showed significant differences between forest and grassland, as well as between forest and shrubland (*P<0.05*). The Margalef index, Shannon-Wiener index, and Pielou index for plant communities in grassland, shrubland, and forest soils all followed similar patterns: shrubland exhibited the highest values, while forest showed the lowest. The Simpson index was highest in the forest (0.94) and lowest in the grassland (0.87).

**Table 2:** Species diversity index of different vegetation type communities.

Vegetation type	R	H	J	S’
Grassland	5.06 ± 0.88a	2.79 ± 0.18a	0.90 ± 0.01a	0.87 ± 0.02a
Shrubland	5.27 ± 0.43a	2.92 ± 0.06a	0.90 ± 0.00a	0.91 ± 0.01a
Forest	4.64 ± 0.92a	2.52 ± 0.20a	0.83 ± 0.01b	0.94 ± 0.00a

Different lowercase letters in the same column indicate significant differences (*P<0.05*); R is the Margalef index, H is the Shannon-Wiener index, J is the Pielou index, and S’ is the Simpson index.

### 3.4 Correlation analysis of soil physicochemical properties, nutrient elements, plant diversity indices, and soil stoichiometric characteristics

**Figure 3:**
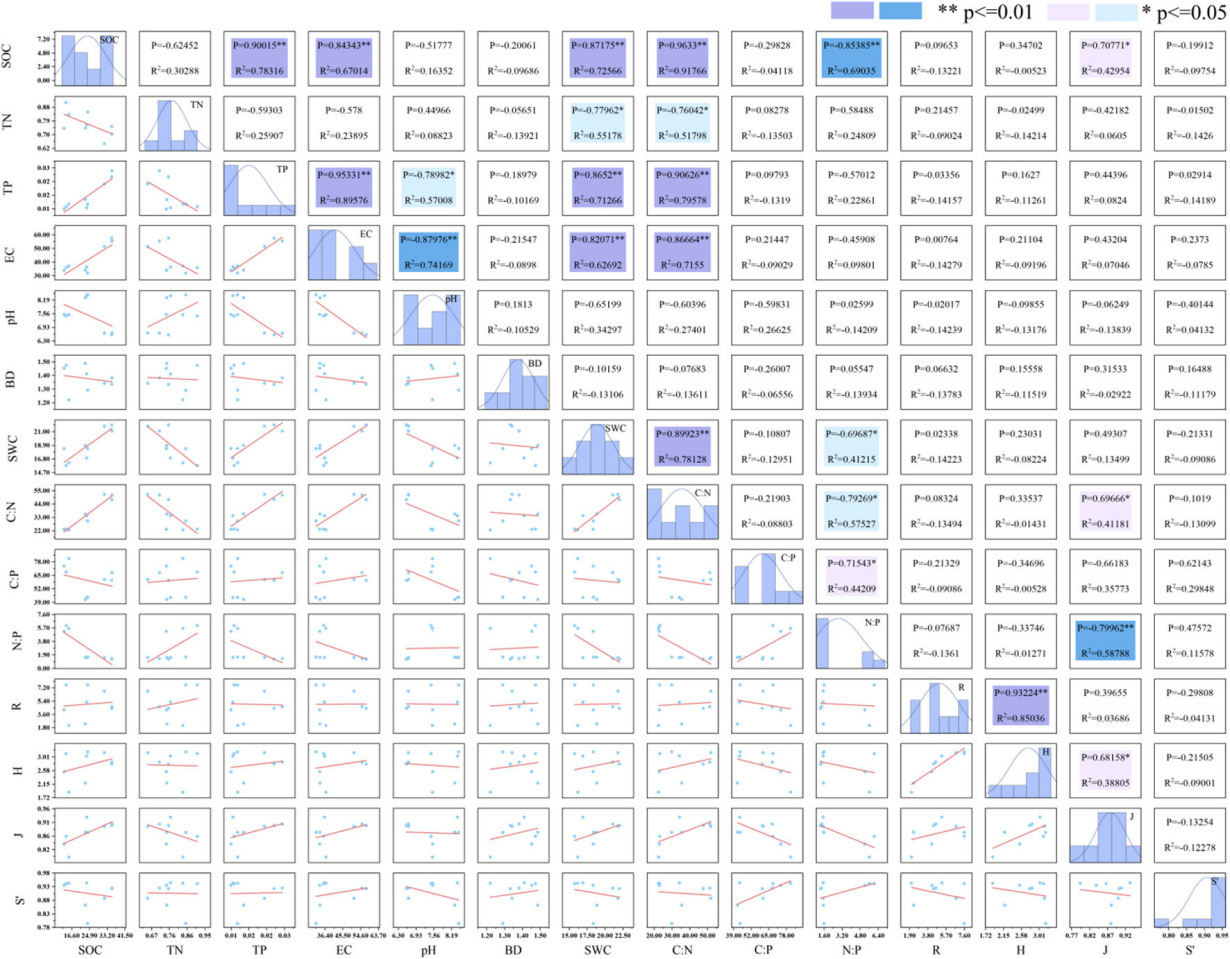
Correlation analysis of soil stoichiometry, nutrient elements, physicochemical properties and plant diversity index. * indicated significant correlation (*P<0.05*), ** indicated significant correlation (*P<0.01*).

As shown in **Figure 3**, significant positive correlations exist among soil C:N, TP, SWC, SOC, and EC indicators, indicating close relationships among nutrients. Soil C:N showed significant negative correlations with soil N:P and TN (*P<0.05*), and a significant positive correlation with the Pielou index (*P<0.05*); Soil C:P and N:P showed a significant positive correlation (*P<0.05*), while N:P exhibited significant negative correlations with SOC, SWC, and the Pielou index (*P<0.05*).

**Figure 4:**
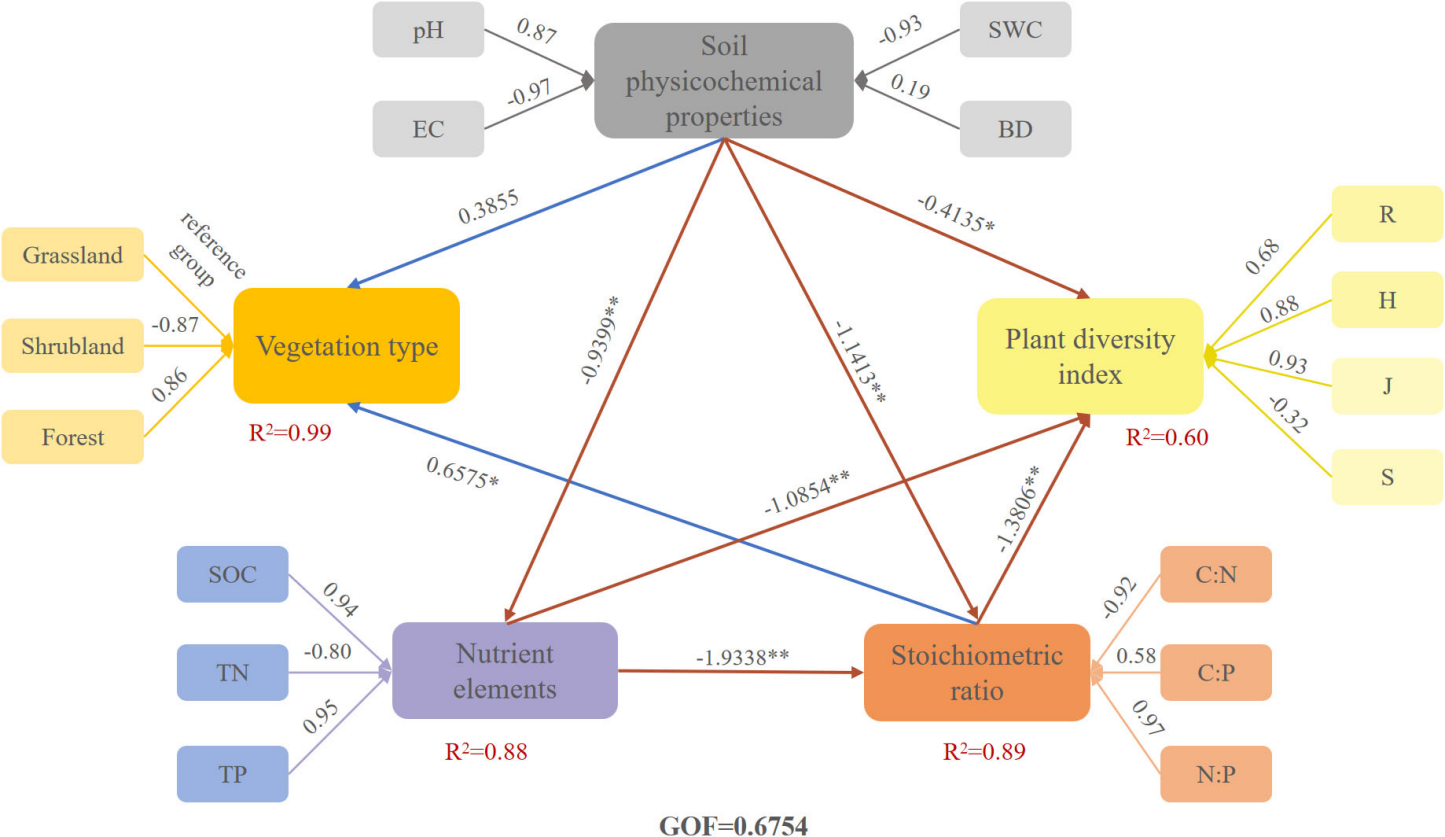
Structural equation models of vegetation type, soil physicochemical properties, nutrient elements, stoichiometric ratio, and plant diversity index. The blue arrow indicates a positive impact, while the red arrow indicates a negative impact. * indicated significant correlation (*P<0.05*), ** indicated significant correlation (*P<0.01*).

Using partial least squares structural equation modeling, we quantified the interaction and influence pathways among soil physicochemical properties, nutrient elements, ecological stoichiometric ratios of carbon, nitrogen, and phosphorus, and plant diversity indices across vegetation types. We evaluated the model reliability and validity using composite reliability (DG. Rho) and average extracted variable (AVE) coefficients; all latent variables showed AVE values above 0.5, indicating good convergent validity. The model’s Goodness-of-Fit (GoF) value was 0.6754, reflecting a good overall fit. Loadings greater than 0.7 for observed variables indicated strong contributions to their respective latent variables. Analysis results are presented in **Figure 4**. Soil physicochemical properties had direct effects on vegetation type, nutrient elements, plant diversity index, and stoichiometric ratios, with path coefficients of 0.3855, -0.9399, -0.4135, and -1.1413. Chemical ratios directly affected plant diversity indices and vegetation types, with coefficients of -1.3806 and 0.6575. Nutrient elements directly influenced plant diversity indices and stoichiometric ratios, with coefficients of -1.0845 and -1.9338. Larger absolute path coefficients indicate stronger effects. Variance explained for vegetation type, nutrient elements, plant diversity index, and stoichiometric ratios was 99%, 88%, 60%, and 89% respectively. The soil physicochemical properties' R^2^ value was 0, showing that other model variables did not significantly impact these properties.

## 4 Discussion

### 4.1 Relationships among soil physicochemical properties, nutrient elements, and stoichiometric characteristics

SOC content varied with vegetation type, influenced by environmental conditions and vegetation composition (Wu et al., 2024). Shrubland vegetation in karst peak cluster depressions exhibits the highest SOC content. This vegetation significantly contributes to SOC accumulation by influencing soil microbial residual carbon content and its relationship with soil physicochemical properties. Typical karst mountain scrubland exhibits the highest oxidized organic carbon content (Shen et al., 2025). This is attributed to the looser soil structure of scrubland compared to other vegetation types, allowing organic matter to accumulate effectively within the soil and migrate vertically downward. Additionally, shrubs produce more decomposable litter layers and possess more developed fine root systems (Huang et al., 2025). These characteristics indicate shrubs’ influence on soil organic carbon. This further demonstrates that shrublands exhibit relatively strong carbon sequestration effects and possess certain carbon sequestration potential in areas affected by rock desertification.TN content in karst peak cluster depression shrublands and forest soils decreases with increasing soil depth (Su et al., 2019). This is attributed to the greater aggregation effect in the surface layer of shrubland and forest soils, where decomposed surface litter is transported downward through the soil profile via water or other media, resulting in more pronounced nutrient return effects. In grasslands, TN content gradually increases with soil depth, while TP content remains relatively stable. This finding contrasts with the results reported by SU et al. for shrublands and forests (Su et al., 2019). This discrepancy may stem from the intense surface runoff characteristic of karst regions, where topsoil and nutrients leach downward through fissures and accumulate in deeper soil layers or crevices, leading to a gradual increase in TN content. The study area's average soil TN and TP contents were 0.77 g·kg^-1^ and 0.01 g·kg^-1^, respectively, both below the national average for terrestrial ecosystems in China (1.88 g·kg^-1^ and 0.78 g·kg^-1^, respectively) (Tian et al., 2010). This further indicates nitrogen deficiency in the study area's soil, characterizing the soil environment as nitrogen-deficient (Ma Jian et al., 2021).

Lower soil C:N ratios correlate with higher mineralization levels and accelerated soil organic matter decomposition rates (Wang et al., 2018), thereby promoting litter decomposition. Compared to the global average C:N ratio of 14.3 (Zhao et al., 2015), the karst peak-and-hollow ecosystem exhibits lower rates of soil organic matter decomposition and mineralization. A soil N:P ratio below 14 indicates relative nitrogen deficiency in this region (Plach et al., 2017). **Figure 3** shows a negative correlation between N:P and TP content, further confirming that soil nutrients in karst peak clusters are primarily nitrogen-limited, followed by phosphorus limitation. Soil C:P is regarded as an indicator of phosphorus mineralization capacity (Wang et al., 2018). The C:P ratio in this study’s soils is lower than China's average (136) (Tian et al., 2010), far below the global average (186) (Zhao et al., 2015). Findings indicate phosphorus is not deficient relative to carbon; however, due to the calcareous nature of karst soils, phosphorus is readily fixed by calcium, limiting the release of available phosphorus. Plants thus face secondary phosphorus limitation with insufficient absorbable phosphate content. This aligns with the region’s tendency toward dual N-P limitation in plants, indicating significant habitat stress.

SOC content in different vegetation types across karst peak clusters and depressions showed a significant positive correlation with SWC. This finding aligns with the conclusions of Luo et al.’s existing research (Luo et al., 2025). Soil moisture plays a critical role in regulating microbial carbon content and metabolic activity (Du et al., 2016), thereby influencing the decomposition rate and pathways of soil organic carbon. Furthermore, variations in soil moisture content driven by different vegetation types further regulate the accumulation efficiency and transformation processes of soil organic carbon pools by influencing soil aggregate stability and pore structure characteristics. In this study, both SWC and SOC both SWC and SOC followed the order: shrubland > grassland > forest. This is because shrubland species generally possess higher biomass and faster growth rates, promoting nutrient accumulation and improving physical properties such as SWC. Similar to previous finding (Gentili et al., 2011), shrub canopy intercepts precipitation, reducing surface runoff and evaporation losses while promoting soil moisture infiltration and storage, thereby influencing soil organic carbon. Grasslands exhibit lower transpiration water consumption, resulting in higher soil moisture content. This study found that shrubland exhibited lower pH values than grassland and forest across all soil horizons, with significant differences observed between shrubland and grassland soils. Similar to the findings of Zukswer et al (Zukswert and Prescott, 2017), the lower pH observed in shrub communities can be attributed to differences in vegetation functional traits. Shrub leaves are smaller and decompose more rapidly after falling. This accelerated decomposition of organic matter leads to the release of acidic compounds, thereby lowering soil pH. Wu (Wu et al., 2020) found that under different vegetation types, vegetation establishment not only directly increases soil nutrient pools by supplying organic matter inputs such as litter, plant residues, and root exudates, but also indirectly drives nutrient transformation and cycling processes by shaping specific soil microbial community structures and functions. Differences in litter quality and root exudates significantly influence microbial composition and activity, thereby regulating key processes such as organic matter decomposition, nutrient mineralization, and immobilization. Collectively, these mechanisms determine the availability and retention of soil nutrients in karst ecosystems.

### 4.2 Relationships between soil physicochemical properties, nutrient elements, and plant diversity

Variations in soil conditions impact plant growth and development, thereby influencing species diversity (Sheng et al., 2015). Research findings indicate that the Simpson index value is highest in forest communities within this region, demonstrating a significant advantage of forest ecosystems in maintaining high levels of biodiversity (He et al., 2025). Research findings reveal that shrub communities exhibit the highest Margalef index values, Shannon-Wiener indices, and Pielou’s evenness indices. This indicates greater species richness, more foliage, and increased litterfall in shrub communities, which can substantially enhance soil organic matter accumulation (Yin et al., 2017). SOC showed a significant positive correlation with the Pielou index (*P<0.01*), indicating that increased SOC promotes the even distribution of plant individuals across species, or that communities with higher evenness favor SOC accumulation. The Margalef index showed a significant positive correlation with the Shannon-Wiener index (*P<0.05*), while the Shannon-Wiener index itself exhibited a significant positive correlation with the Pielou index (*P<0.01*). This suggests a clear positive relationship between soil organic carbon and plant diversity, indicating that soil organic carbon is a key factor in maintaining soil health and plant diversity (Sheng et al., 2015). Soil pH, BD, SWC, EC, and plant diversity exhibit certain variations across different vegetation types, indicating a close interactive relationship between species diversity and soil physicochemical properties. Changes in SWC and EC are among the primary factors influencing understory plant diversity (Jun-shan et al., 2023). However, this study found no significant correlation between SWC, EC, and plant diversity. This discrepancy may be attributed to climatic conditions and soil types. Furthermore, previous studies have demonstrated that TN significantly influences species distribution and diversity. However, no significant correlation was observed in this study. This discrepancy may stem from the complex and heterogeneous habitats within karst ecosystems, where diverse environmental factors interact synergistically or antagonistically. Such interactions could either amplify or attenuate the effects of individual ecological factors, potentially masking the influence of TN behind other more dominant variables (Shao et al., 2012). Future research should integrate additional environmental factor variables to further explore their combined effects.

### 4.3 Relationship between soil stoichiometric characteristics and plant diversity

The stoichiometry of soil C, N, and P is considered a proxy for soil nutrient cycling and characterizes the availability of soil N and P to plants in grasslands and forests (Wang et al., 2020). Ecological stoichiometric traits are widely applied in diagnosing plant community stability and growth-limiting elements. Ecological chemometrics plays a significant driving role in plant community diversity. C is a primary element influencing plant growth (Heyburn et al., 2017). Compared to other nutrients, it exhibits high and stable concentrations, yet C is not the key limiting factor for plant growth. In this study, the Pielou index showed a significant positive correlation with C:N (P<0.01), a significant negative correlation with N:P (*P<0.01*), and a negative correlation with TN. This indicates that soil SOC and N:P content exert more pronounced effects on plant community species diversity by altering soil properties that influence plant growth, thereby impacting species diversity. The soil N:P ratio further confirms that soil N is the primary limiting nutrient for plant distribution in karst peak cluster depressions, where species composition and distribution are susceptible to the influence of soil N:P dynamic equilibrium.

### 4.4 Relationships between soil physicochemical properties, nutrient elements, and stoichiometric characteristics of different vegetation types and plant diversity

Vegetation is one of the primary factors influencing soil nutrient chemistries (Tao et al., 2020). Different species exhibit distinct biological characteristics, leading to significant variations in their impact on soil nutrients. Consistent with previous findings (Joshi and Garkoti, 2023), the physicochemical properties of karst peak cluster depression soils exert a direct positive effect on vegetation types, with a path coefficient of 0.3855. Soil physicochemical properties exert a direct negative effect on nutrient elements. This occurs because increased SWC promotes nitrogen leaching, leading to nutrient loss. Alternatively, excessively high or low pH values may cause phosphorus fixation or leaching, thereby reducing its availability (Zhang et al., 2023). Soil pH is a critical factor influencing nutrient availability and chemical transformations, directly affecting nutrient existence states, conversion processes, and availability. SWC directly impacts nutrient mobility and litter decomposition rates. These factors collectively explain the negative correlation between soil physicochemical properties and nutrient content. This study demonstrates that soil physicochemical properties exert a direct negative effect on species diversity indices. This occurs because variations in soil physicochemical properties influence soil microbial activity, soil organic matter decomposition rates, and plant root growth and nutrient uptake capacity. Consequently, they affect nutrient content and availability in the soil, thereby directly negatively impacting plant diversity indices. Similar to the findings of Lu et al., the results indicate that soil physicochemical properties exert a direct negative effect on the stoichiometric ratio (Lu et al., 2023), with a path coefficient of -1.1413.

This study found that SOC is the primary soil factor influencing understory plant diversity, while N and P are important soil factors that indirectly affect understory plant diversity by regulating soil properties (Meng et al., 2023). Soil C:N, C:P, and N:P ratios vary across different vegetation types and their substrates (Zechmeister-Boltenstern et al., 2015). In this study, vegetation type exerted a direct influence on soil C:N, C:P, and N:P ratios. These findings collectively demonstrate that differences in soil physicochemical properties, nutrient elements, and stoichiometric characteristics across vegetation types directly or indirectly shape plant community diversity. The findings contribute to developing sustainable management strategies and long-term soil management guidelines. They also provide a more detailed understanding of the intrinsic relationships between soil plant diversity indices and environmental factors under different vegetation types. This knowledge enables effective management of ecosystems around FAST and karst peak clusters and depressions to respond to natural changes.

## 5 Conclusion

This study investigated the relationships among soil chemical characteristics, physicochemical properties, nutrient elements, and plant diversity indices across different vegetation types (grassland, shrubland, forest) in karst peak cluster depressions. The findings indicate:

1. Shrubland vegetation in the study area exhibited the highest SOC content, demonstrating potential for carbon sequestration in rock desertification regions. Shrublands can serve as a priority vegetation type for carbon sink restoration. Soil N deficiency was prevalent, with N-limiting conditions dominating nutrient availability, followed by P limitation.
2. Forest communities exhibited the highest Simpson dominance index, while shrub communities showed the highest Margalef index, Shannon-Wiener index, and Pielou index. SOC correlated significantly positively with the Pielou index.
3. Soil SOC, TN, and TP content exerted a more pronounced influence on plant community species diversity in the study area, with species composition and distribution being susceptible to soil N:P dynamic equilibrium.
4. Partial Least Squares Structural Equation Modeling analysis revealed that soil physicochemical properties exerted a direct positive effect on vegetation type, while exerting a direct negative effect on soil nutrient elements, plant diversity indices, and stoichiometric ratios.

## Data Availability

The original contributions presented in the study are included in the article/supplementary material. Further inquiries can be directed to the corresponding author.
